# Supplementary material for: Small molecule binding to surface-supported single-site transition-metal reaction centres
Source: Nat Commun. 2022 Dec 1;13:7407. doi: 10.1038/s41467-022-35193-6 (PMC9715722; doi:10.1038/s41467-022-35193-6)
Supplement: Supplementary file 1 — Supplementary Information [file 41467_2022_35193_MOESM1_ESM.pdf]

Supplementary Information for

"Small molecule binding to surface-supported single-site reaction centers"

M. DeJong[1,2], A. J. Price[3], E. Mårsell[2], G. Tom[1,2], G. D. Nguyen[2], E. R. Johnson[3*], and S. A. Burke[1,2,4 *]

1. Department of Physics & Astronomy, University of British Columbia, Vancouver, V6T 1Z1, Canada
2. Stewart Blusson Quantum Matter Institute, University of British Columbia, Vancouver, V6T 1Z4, Canada
3. Department of Chemistry, Dalhousie University, Halifax, B3H 4R2, Canada
4. Department of Chemistry, University of British Columbia, Vancouver, V6T 1Z1, Canada

* corresponding authors: Sarah Burke saburke@phas.ubc.ca; Erin Johnson Erin.Johnson@Dal.Ca

Supplementary Note 1: Voltage pulse induced switching and dissociation of C_2H_4 prebond and bond structures

Bias pulses of V=-0.7V for t=200ms applied ~2.0nm away from the C_2H_4 prebond and bond terminations induced switching between bond and prebond structure (Supplementary Fig. 1a-c). Pulsing under the same conditions within ~0.5nm from the C_2H_4 bond node resulted in complete dissociation of the C_2H_4 bond motif leaving a single C_2H_4 molecule ejected and bare Fe-tpy active site (Supplementary Fig. 1d, lower molecule and Supplementary Fig. 1e, upper molecule). Controlled switching between CO prebond to bond motif was not achieved under these pulsing conditions, likely due to the instability of the CO prebond motif and low diffusion barrier of CO on Ag(111).

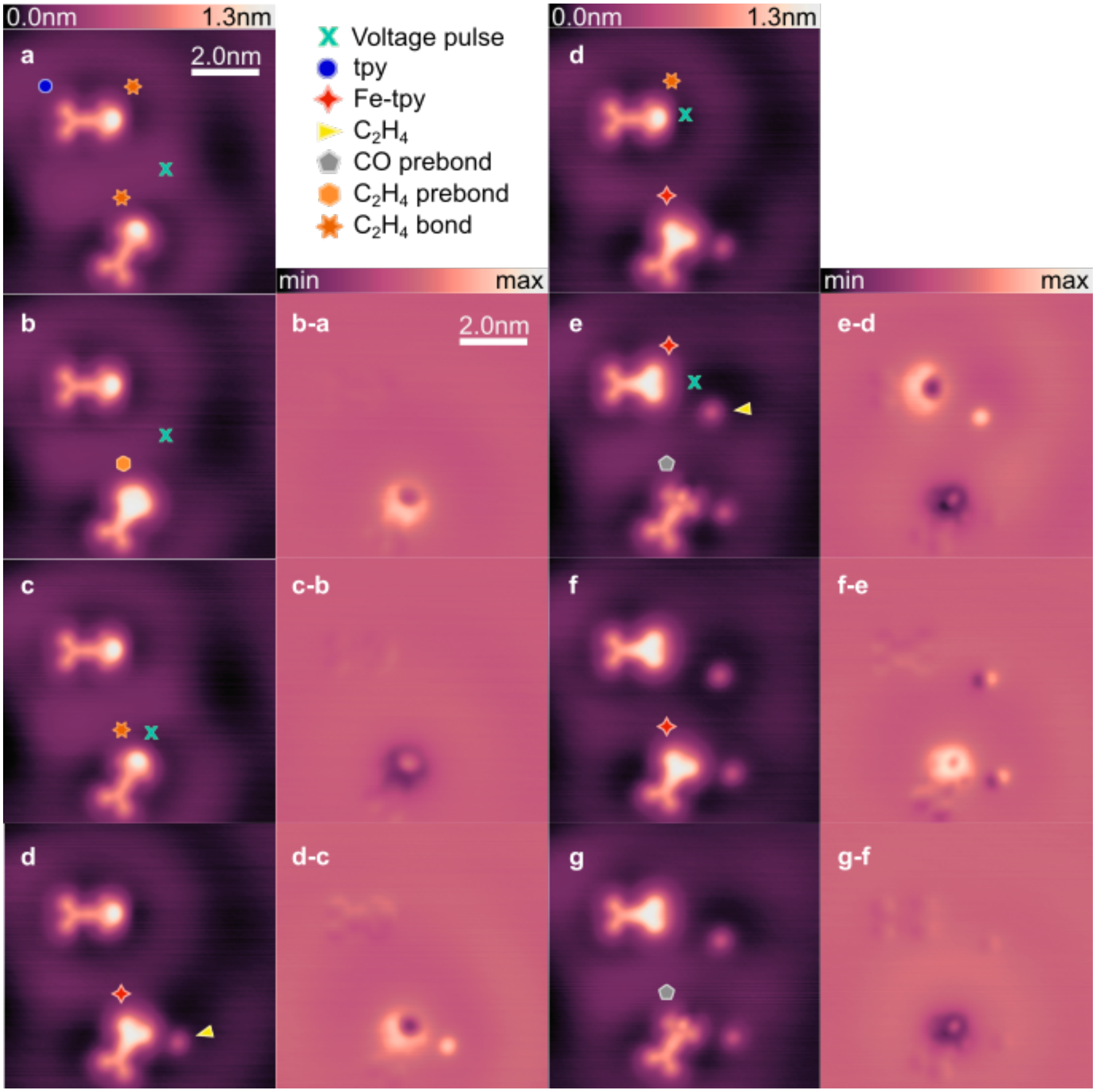

Supplementary Figure 1: Series of topographs acquired consecutively (a-g) showing the same area of a sample before and after voltage pulses of V=-0.7V for t=200ms were applied at various locations in the frames (pulse locations marked with teal 'x'). Indicated pulses were applied after frame acquisition was complete. Difference topographs, (b-a) through (g-f), highlight structural changes induced by pulsing such as the switch from C_2H_4 bond to C_2H_4 prebond visible in (b-a), and the reverse occurring in (c-b). Complete dissociation of the C_2H_4 bond is achieved in (d-c) for the lower molecule, and in (e-d) for the upper molecule. Frame (d) is repeated in both columns for ease of comparison. Scan-induced changes to node structure are apparent in (g-f) where the lower molecule changed from an Fe-tpy termination to a CO prebond without bias pulse provocation. V=-25mV and I=50pA for all frames.

Supplementary Note 2: Additional STS measurements with locations and dI/dV maps

Two different measurements for the CO bond (Supplementary Fig. 2b), and C_2H_4 bond (Supplementary Fig. 2c) reinforce the peak tunneling positions at -0.16V and -0.30V, respectively. Although the line spectra in Supplementary Fig. 2a-c are scaled by the setpoint parameters, the magnitude of CO bond 1 dI/dV is noticeably less than CO bond 2 dI/dV in Supplementary Fig. 2b. This is expected since the set point for the CO bond 1 grid was -280mV, much closer to the state at -0.16V than the setpoint for the CO bond 2 grid at +300mV.

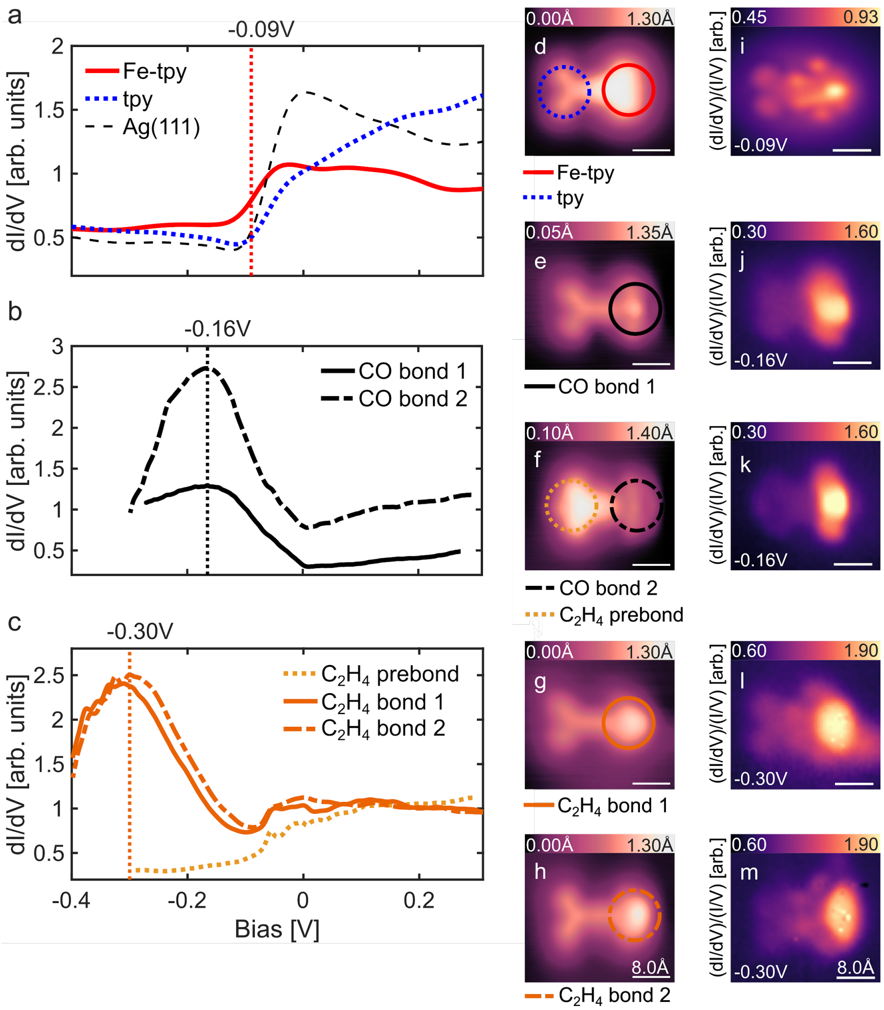

Supplementary Figure 2: Average dI/dV line spectroscopy plots (a-c), STM topographs (d-h), and normalized dI/dV bias maps (i-m) that correspond to each topograph directly to the left. STM topographs (d-h) show the areas corresponding to 185 spatial pixels averaged over to generate the STS shown (solid and dashed circles in topographs). The bias maps (i-m) show the spatial distribution of electronic states at the resonance positions. Bias maps for the CO bond at -0.16V (j,k right) show a maximum intensity circular region that spreads out vertically, whereas the C_2H_4 bond at -0.30V (l,m) show an evenly distributed oval prominent feature. STM topograph scanning parameters: -100mV/20pA (d), -20mV/100pA (e), 20mV/20pA (f), and 20mV/50pA (g,h). Grid setpoint parameters: -2V/1nA (i), -280mV/1nA (j), 300mV/1.5nA (k), 400mV/1.5nA (l,m).

Supplementary note 4: Intervention steps and coarse reaction statistics

The "anneal & quench" approach used required annealing for 5 minutes at a set temperature and then returning to 4.2K for imaging. Sequential annealing steps after initial dosing of ethylene (during which the temperature rose to 11.6K) were incremented by 5K and the resulting changes to the surface observed. In the example shown, after annealing to 30K, a tip change necessitated moving to a new location which had little ethylene present – perhaps due to shadowing effects from the tip during the initial dosing – and a second dose of ethylene (2nd grey block) was added and annealing steps resumed at 30K. These are shown together since the sample had already been exposed to the prior annealing potentially leading to some changes. All data shown in main text originates from this run. Additional runs primarily annealing at or above 30K showed similar results.

The plot below also shows coarse statistics, based on counting by eye, for ethylene observed in different structures. CO is omitted for clarity. While there are not sufficient statistics to expect quantitative reliability, the general trend of increasing pre-bond to bond conversion and observation of "other products" with increased annealing indicate the direction of reaction progression.

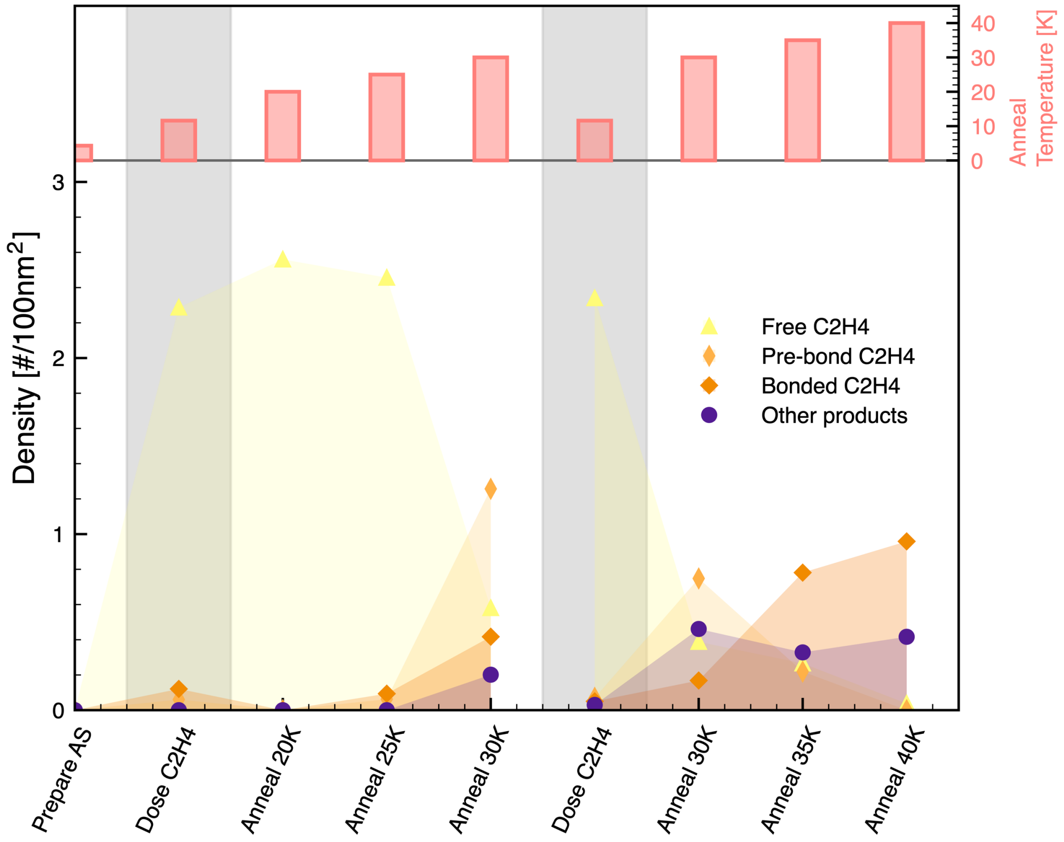

Supplementary figure 3: Schematic of annealing steps and coarse statistics (measured at 4K following each anneal step) for ethylene species.

Supplementary note 5: ncAFM amplitude calibration

Calibration of the ncAFM oscillation amplitude was performed for the qPlus sensor used for all measurements presented in this work following the method described in Simon et al.[1] by increasing the oscillation amplitude while maintaining constant tunnelling current feedback and measuring the change in z position. Past ~50mV excitation amplitude, the z vs. amplitude relationship becomes linear and an amplitude-voltage conversion factor of 0.0039nm/mV was found. Typical excitation amplitudes of 50mV were used during most image acquisition corresponding to a free oscillation amplitude of ~2Å.

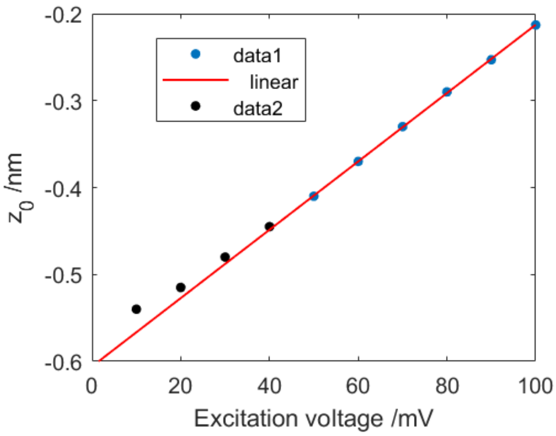

Supplementary figure 4: ncAFM amplitude calibration performed under constant tunnelling current feedback conditions. Around 40-50mV excitation voltage, the z vs. excitation amplitude curve becomes linear providing a calibration of the oscillation amplitude of the tip. Data1 (blue points) shows the data in a linear regime used for the fit; Data2 shows deviation from linear at small amplitudes as expected.

Supplementary note 6: Bader analysis

Bader atomic charges were computed from the B86bPBE electron densities using the Yu-Trinkle algorithm[2] implemented in the critic2[3] program.

Supplementary Table 1: Bader charge analysis for Fe-tpy species

Species	Configuration	Fe charge	Reactant change
Fe-tpy	-	0.818	-
CO	Vertical/pre-bond	0.899	-0.522
CO	Horizontal/bond	0.893	-0.178
C_2H_4	Horizontal/pre-bond	0.877	-0.371
C_2H_4	Vertical/bond	0.830	-0.403

Note that in previous work, Fe coordinated to a similar terpyridine ligand showed signatures of Fe^{2+} in NEXAFS, corresponding to a Bader charge of ~0.8e$^-$. As discussed in the main text, we expect iron in each of these species to be in the nominally 2+ state (recognizing that in proximity

to the Ag(111) surface and some hybridization a pure oxidation state picture is not entirely accurate)

Supplementary note 7: Table of binding energies from DFT calculations of Fe-tpy

Supplementary Table 2: binding energies of optimized structures with breakdown of base functional and dispersion contributions to total. Binding energies for CO and C_2H_4 are relative to Fe-tpy.

**Species**	**Configuration**	**Binding energy (kcal/mol)**	**Base functional contribution (kcal/mol)**	**Dispersion contribution (kcal/mol)**
Fe-tpy	-	78.3	21.3	57.0
CO	Vertical/pre-bond	17.6	10.5	7.1
CO	Horizontal/bond	48.6	42.0	6.6
C_2H_4	Horizontal/pre-bond	19.9	8.1	11.8
C_2H_4	Vertical/bond	48.6	42.0	6.6

Supplementary note 8: Geometries of optimized DFT structures of Fe-tpy

Key bond lengths for each structure calculated by DFT are given in the tables below as well as additional side-views to better visualize each of the optimized structures and relative position to the substrate surface. Full structure files are available via OSF at https://osf.io/2tke6/ DOI 10.17605/OSF.IO/2TKE6

Supplementary Table 3: bond distances in Å for CO structures

	C-O	Fe-C	Ag-C	Fe-O
CO	1.14	--	--	--
CO pre-bond	1.22	1.88	2.12	2.13
CO bonded	1.18	1.75	--	--

Supplementary Table 4: bond distances in Å for C_2H_4 structures

	C=C	Fe-C1	Fe-C2
C_2H_4	1.33	--	--
C_2H_4 pre-bond	1.37	2.13	2.13
C_2H_4 bonded	1.43	2.00	2.12

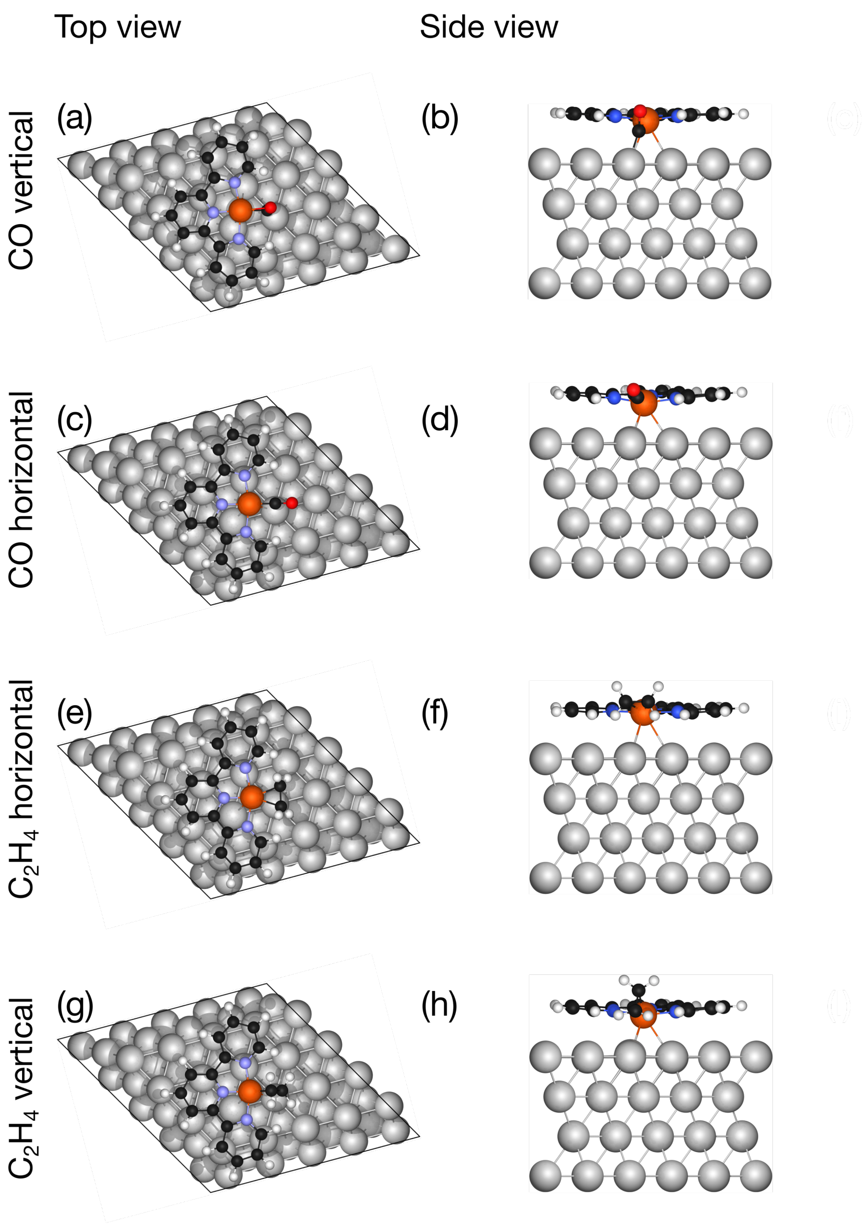

Supplementary figure 5: Left column: top-view (as shown in main text), and right column: side-view of each of the four optimized structures CO-vertical (a,b), CO-horizontal (c,d), C_2H_4-horizontal (e,f), and C_2H_4-vertical (g,h)

Supplementary note 9: Orbital energies of bare and CO-coordinated complex

To compare to a Ligand-Field model, gas-phase (RO)B3LYP[4,5] calculations with a mixed basis set using 6-31+G* for Fe, N, O and 6-31G* for C, H were performed using Gaussian [6]on the bare Fe-tpy site and CO bond structure. The bare Fe-tpy vaguely resembles a square planar coordination geometry, however, already, even without a surface, we can see that the order of the orbitals are altered by the asymmetric coordination environment. The LUMO+1 also presents

a distinct feature unique to the asymmetric environment containing a mixed dx^2+p_x character. The HOMO and LUMO also consist of π-orbitals with weight distributed across the Fe and ligand, and are nearly degenerate with the dz^2 orbital, which likely corresponds to the prominent feature seen in STM and STS (in part due to the directionality and strongly protruding nature of the electron density). These closely spaced energy levels lead to the observed triplet ground state, consistent with previous reports of tpy and similar ligands providing a weak ligand field. In contrast, the CO bonded to Fe leads to a substantial gap between the d-orbitals and the π states resulting in a singlet ground state. Two of the d-orbitals (HOMO-2, HOMO-3) and one of the π-orbitals (LUMO) participate in the CO bonding.

The orbital character here may provide a helpful framework and highlights some of the unique features of the orbitals owing to the conjugated ligand and asymmetric environment. However, interaction with the silver substrate, which is neglected in these gas phase calculations substantially changes the orbital energy levels, and this should not be taken as quantitative or a complete representation of the surface-bound system.

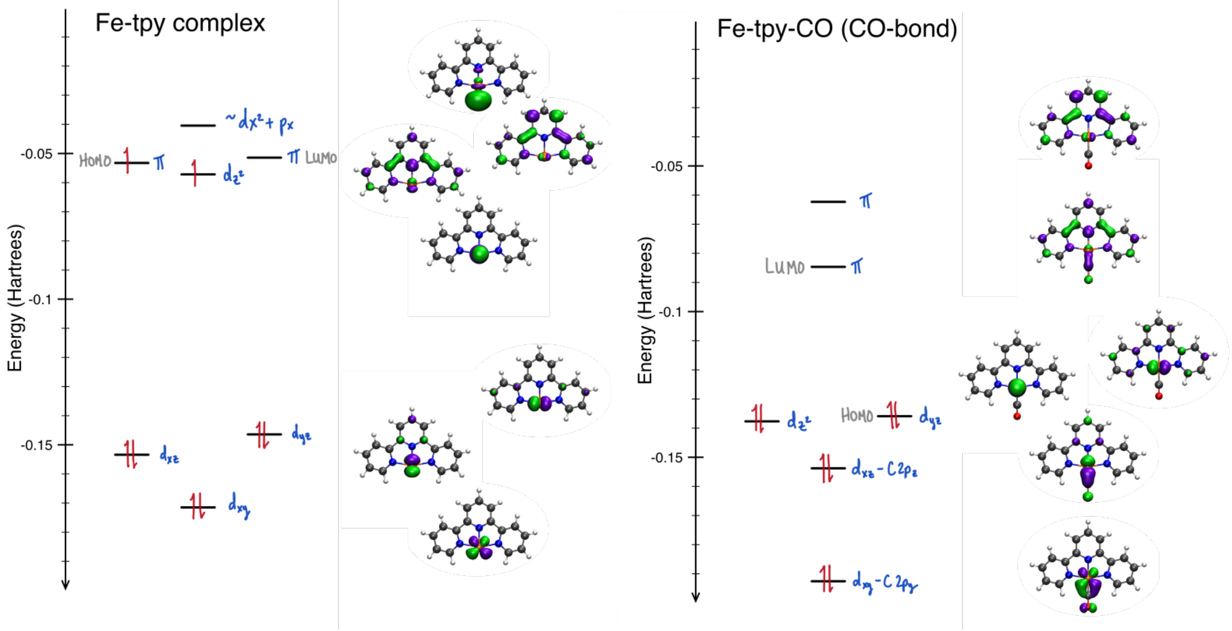

Supplementary figure 6: Orbital energy level sketches based on gas-phase calculations of the nascent Fe-tpy complex (left) and CO bond structure (right), with approximately assigned orbital character. Both complexes include two π-orbitals involving the tpy ligand and five d-orbitals.

Supplementary note 10: nc-AFM simulations at different tip heights

As described in the methods, nc-AFM simulations were performed using the point-probe model developed by Hapala et al.[7] As an accurate tip height is not easily obtained from the experiments, simulations were performed at different heights, shown in Supplementary Fig. 6 below with additional cases posted to OSF at https://osf.io/2tke6/ DOI 10.17605/OSF.IO/2TKE6 with a tip oscillation amplitude of 2Å corresponding to the experimental parameters. The closest

match to the experiment, 16.3Å or 6.3Å between the tip and molecule plane, was used in the main text. Note that this height is reasonable given a tunnelling feedback was used. At smaller distances additional details are observed that are not seen in the data, and at these small distances distortions due to relaxation are often prominent; at larger distances intramolecular features are lost indicative of sampling longer-range interactions.

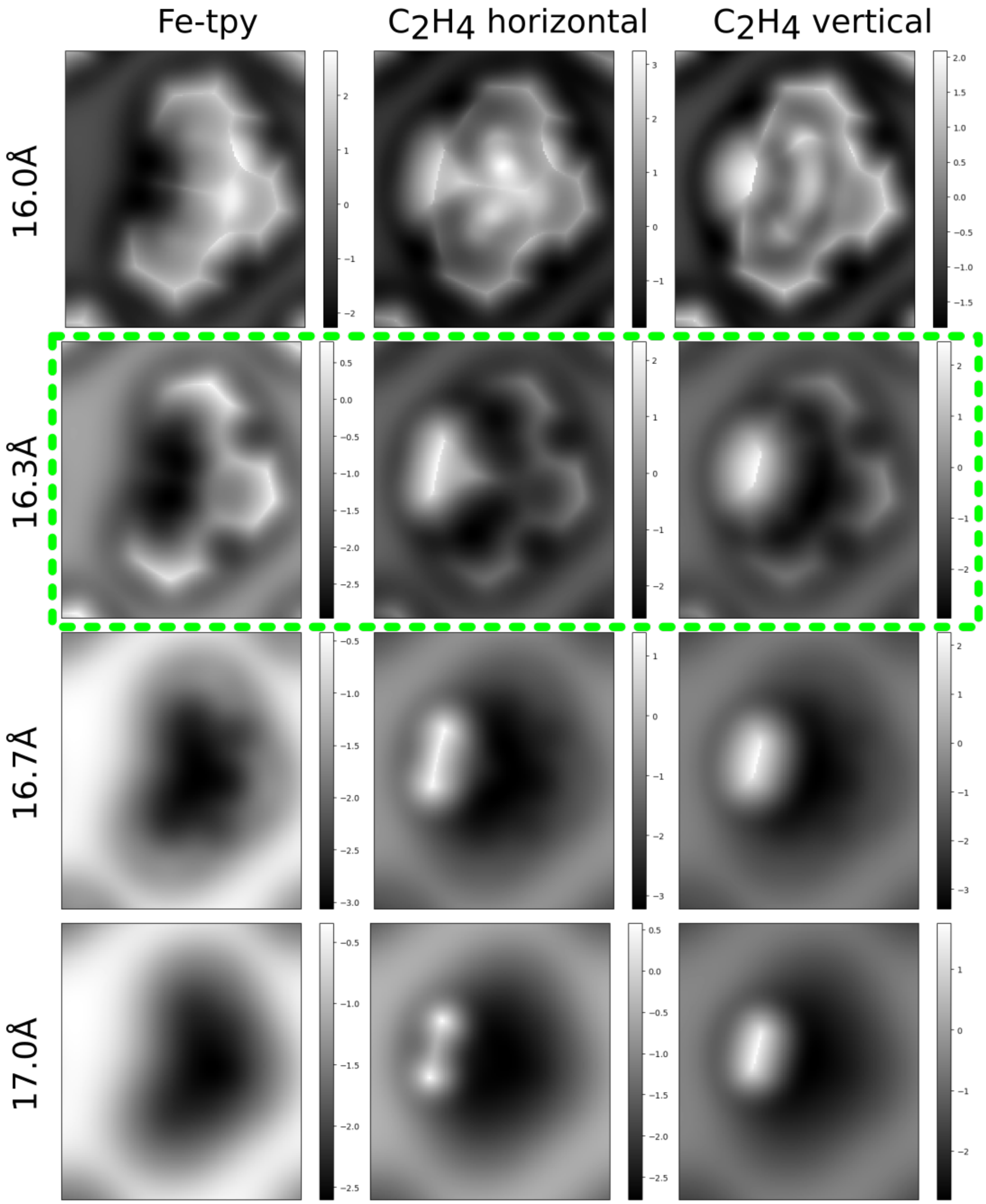

Supplementary figure 7: sampling of ncAFM simulations at different heights. Intensity scale is the un-filtered Δf. Laplace filtered images are shown in the manuscript to match the data processing.

Supplementary References:

1. Simon, G. H., Heyde, M. & Rust, H.-P. Recipes for cantilever parameter determination in dynamic force spectroscopy: spring constant and amplitude. *Nanotechnology* 18, 255503 (2007).

2. Yu, M. & Trinkle, D. R. Accurate and efficient algorithm for Bader charge integration. *J Chem Phys* 134, 064111 (2011).

3. Otero-de-la-Roza, A., Johnson, E. R. & Luaña, V. Critic2: A program for real-space analysis of quantum chemical interactions in solids. *Comput Phys Commun* 185, 1007–1018 (2014).

4. Becke, A. D. Density-functional thermochemistry. III. The role of exact exchange. *J Chem Phys* 98, 5648–5652 (1993).

5. Lee, C., Yang, W. & Parr, R. G. Development of the Colle-Salvetti correlation-energy formula into a functional of the electron density. *Phys Rev B* 37, 785–789 (1988).

6. Frisch, M. J. *et al.* Gaussian 09 Revision B.01.

7. Hapala, P. *et al.* The mechanism of high-resolution STM/AFM imaging with functionalized tips. *Phys Rev B* 90, 085421 (2014).
